# Health and well-being in competitive adolescent distance runners: health problems, training load and psychosocial responses to injury (PhD Academy Award)

**DOI:** 10.1136/bjsports-2022-106608

**Published:** 2023-05-26

**Authors:** Robert H Mann

**Affiliations:** Children's Health and Exercise Research Centre, Department of Public Health and Sport Sciences, University of Exeter, Exeter, Devon, UK

**Keywords:** Athletic Injuries, Pediatrics, Qualitative Research, Running, Epidemiology

## What did I do?

The main aim of my PhD thesis was to describe the extent of the injury and illness problem in competitive adolescent (13–18 years old) distance runners (800 m up to 10 000 m, including the steeplechase). My thesis also: (1) examined the validity of session rating of perceived exertion (sRPE), to quantify internal training load, and (2) explored psychosocial responses to running-related injury (RRI).

## Why did I do it?

Despite the health benefits and popularity of distance running,^1^ research in adults indicates that participation can be associated with adverse health outcomes, such as RRI.^2^ Research on this topic in adolescents is limited, despite the fact that distance running is regularly cited as one of the top three most popular sports for adolescents in England. Thus, research assessing the extent of health problems (RRI and illness), and psychosocial responses to RRI, was necessary in this population. As a practicing youth endurance coach, I was also determined to provide practical tools to support adolescent distance runners in their training, while creating opportunities to integrate the ‘athlete voice’ into broader discussions (eg, policy level) and safeguard the health and well-being of youth athletes.

## How did I do it?

A total of four articles were included in my thesis, related to three separate studies. The first study examined the effects of measurement timing and concurrent validity of sRPE.^3^ This involved 15 (3 women) competitive adolescent distance runners completing a laboratory visit (including measurement familiarisation and a two-part incremental treadmill test), followed by a 2-week mesocycle of training. All training was recorded using a heart rate monitor and a self-report logbook.

The second study used a mixed-methods study design, whereby 113 (64 women) competitive adolescent distance runners across England completed an online questionnaire to generate initial insight into the epidemiology of RRI and to assess training practices.^4^ Based on questionnaire responses, semi-structured interviews were then used to explore psychosocial responses to *serious RRI* (>28 days to 6 months of time loss) in a subsample of athletes. A total of 19 (12 women) athletes volunteered to participate, whereby each interview was split into two main sections—(1) background information and (2) injury experiences—and a thematic analysis approach was used to analyse interview transcripts.^5^

The third study was a 24-week prospective cohort study, throughout which 136 (73 women) competitive adolescent distance runners across England self-reported health problems and training practices on a weekly basis.^6^ Data collection took place between May and October in 2019, with all health problems being recorded using the Oslo Sports Trauma Research Center Questionnaire on Health Problems.^7^

## What did I find?

In study one, it was found that sRPE provides a valid measure of internal training load in competitive adolescent distance runners, irrespective of whether reported 0-min, 15-min or 30-min post-session completion.

**Figure 1:**
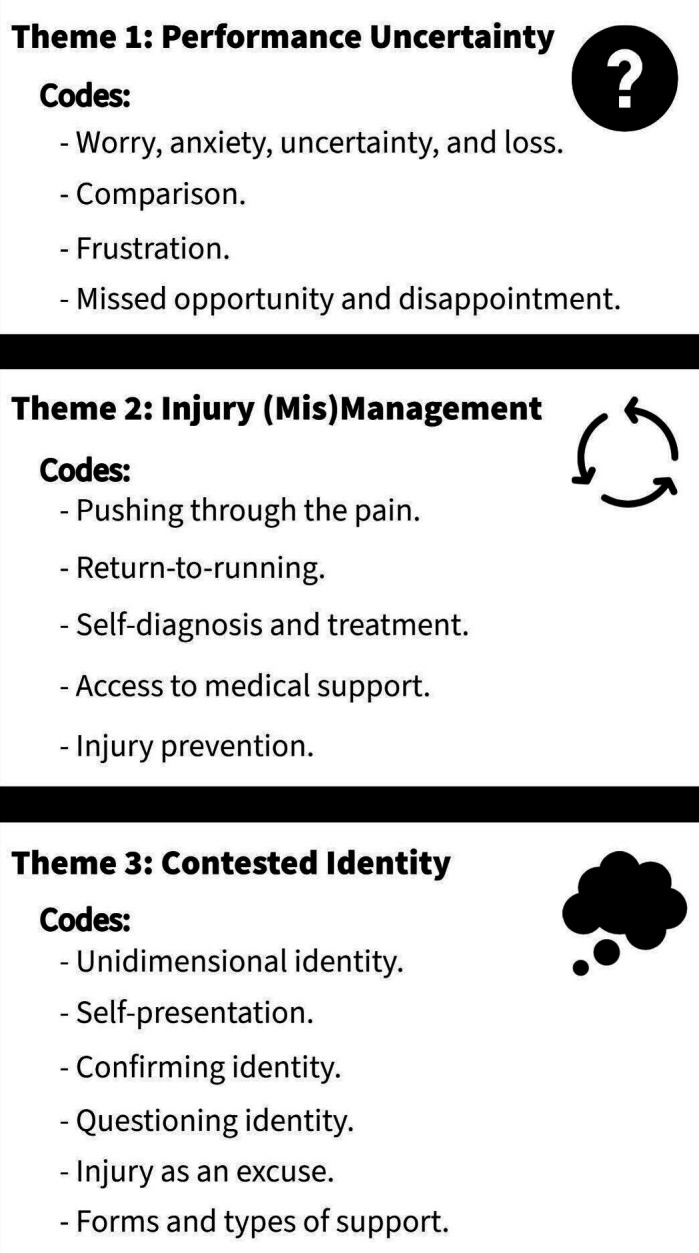
An overview of the themes developed throughout the thematic analysis process completed as part of ‘study two,’ in addition to each of the associated codes.

The second study initially indicated that RRI is common in competitive adolescent distance runners (eg, incidence proportion for ‘all RRI’ = 68%) and that a large proportion of athletes (58%) self-reported a high level of specialisation. In the qualitative part of this study, analysis led to the development of three themes (figure 1) and an overarching understanding that RRI acts to destabilise the athletic identity of competitive adolescent distance runners, as a psychosocial recovery outcome. The three themes—(1) *performance uncertainty*, (2) *injury (mis)management* and (3) *contested identity*—support several theoretical relationships proposed in the integrated model of response to sport injury,^8^ whereby each theme can be viewed as a psychosocial response to *serious RRI*.

The key findings from the third study were that: (1) the incidence of RRI per 1000 hours of exposure was markedly higher when compared with similar samples of adolescent endurance athletes; (2) at any time, 24% of athletes reported a health problem, with 11% having experienced a health problem that had a substantial negative impact on training and performance; (3) female athletes reported noticeably more illness compared with male athletes, including higher prevalence, incidence, time loss and severity; (4) regardless of sex, the most burdensome health problems included lower leg, knee and foot/toes injuries, alongside upper respiratory illnesses; and (5) the mean weekly prevalence of time loss was relatively low, irrespective of health problem type or sex.

## What is the most important practical finding?

Based on the last key finding from the third study, it is feasible that a large proportion of health problems did not cause athletes to miss training or competition. This implies that competitive adolescent distance runners could be training and/or competing while also experiencing a health problem—acknowledged as a ‘silent issue’ that needs to be addressed and supported by further research.

When combining the results from this thesis with other relevant literature, it becomes clear that measures aimed at reducing the risk of RRI in competitive adolescent distance runners need to be developed and integrated within broader ‘health promotion’ initiatives.

## Data Availability

No new data was generated for this article.
